# Supplementary figures and images for: Single-cell transcriptomics reveals immunosuppressive microenvironment and highlights tumor-promoting macrophage cells in Glioblastoma
Source: PLoS One. 2025 Apr 7;20(4):e0312764. doi: 10.1371/journal.pone.0312764 (PMC11975071; doi:10.1371/journal.pone.0312764)

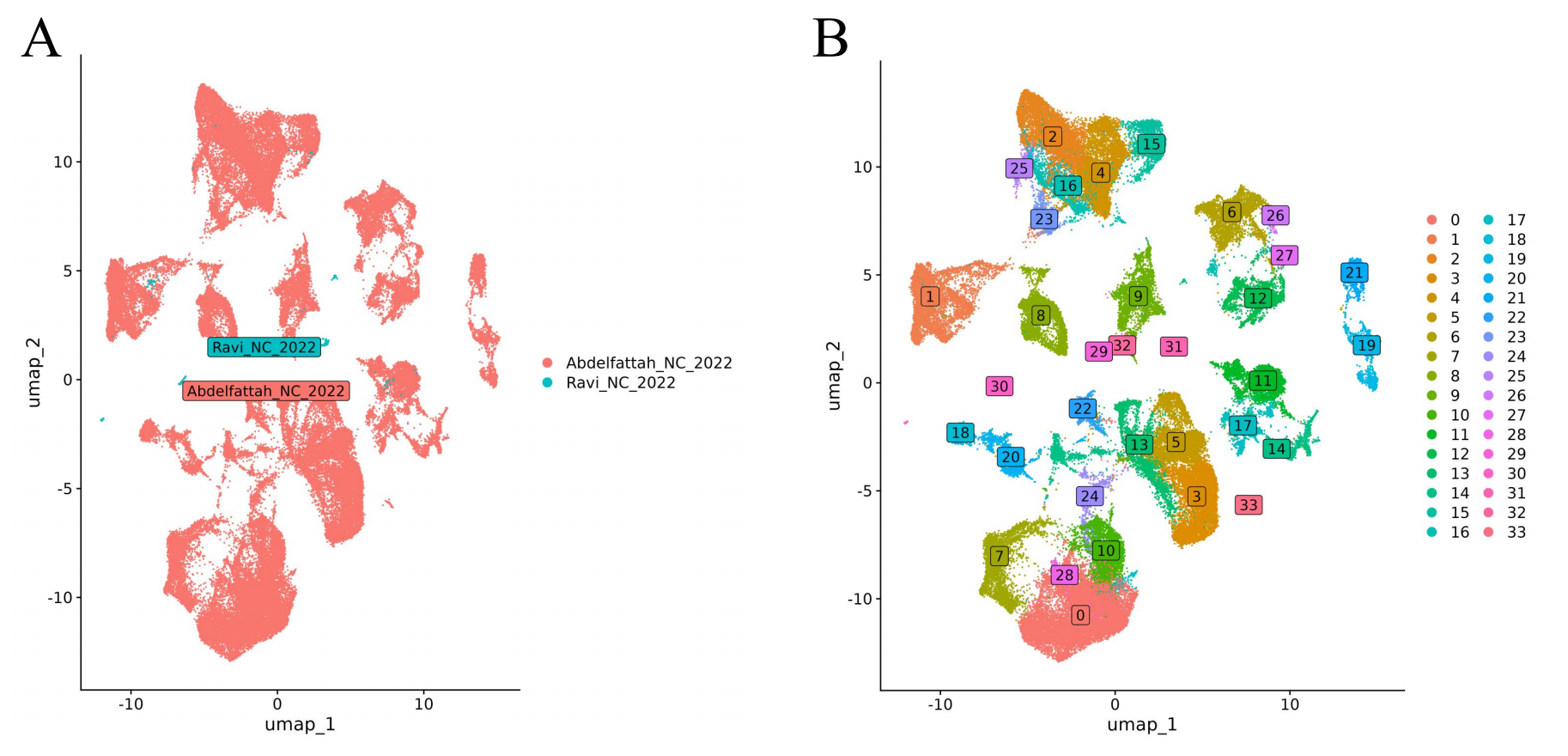

Supplement: S1 Fig — (A) UMAP plots of malignant glioma cells identify in this study, with each cell color coded to indicate the associated dataset. (B) UMAP plots of malignant glioma cells identify in this study, with each cell color coded to indicate the associated clusters. (PNG) [file pone.0312764.s001.png]

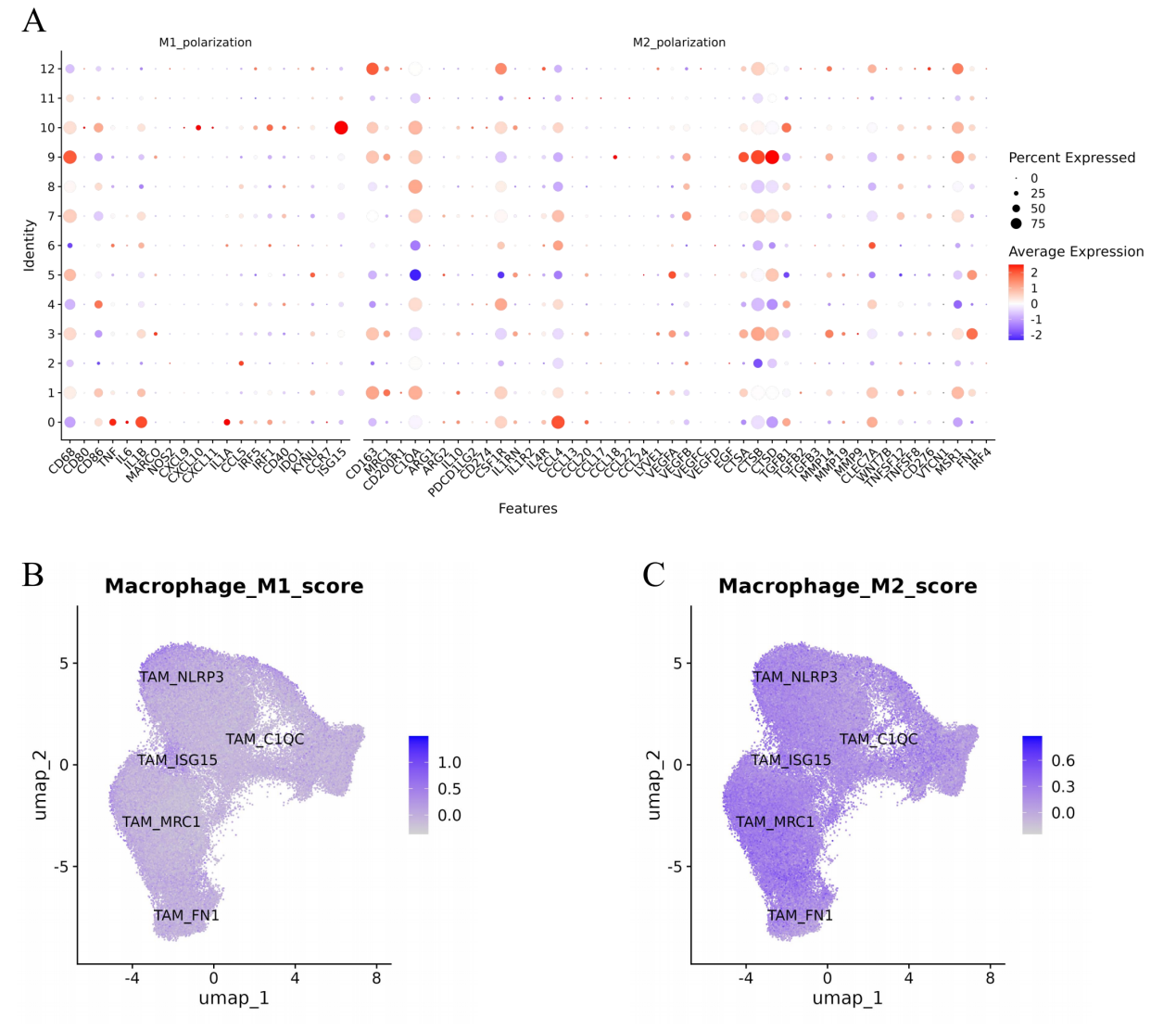

Supplement: S2 Fig — (A) Dot plot showing the average expression of highlighted M1-like and M2-like genes in all TAM clusters. (B) FeaturePlot displayed the M1 polarization scores in five TAM cell subtyps. (C) FeaturePlot displayed the M2 polarization scores in five TAM cell subtyps. (PNG) [file pone.0312764.s002.png]
